# Associations between serum total bilirubin and overactive bladder from the National Health and Nutrition Examination Survey

**DOI:** 10.3389/fendo.2024.1421426

**Published:** 2025-01-14

**Authors:** Bo Jiang, Cunbao Ling, Binbin Dong, Xiaoming Lu, Yadong Liu

**Affiliations:** ^1^ Department of Urology, Affiliated Yancheng Hospital, School of Medicine, Southeast University, Yancheng, Jiangsu, China; ^2^ Department of Urology, Affiliated ZhongDa Hospital of Southeast University, Nanjing, Jiangsu, China; ^3^ Department of Urology, Yancheng Third People’s Hospital, Yancheng, Jiangsu, China; ^4^ School of Basic Medical Sciences, Jiangsu Vocational College of Medicine, Yancheng, Jiangsu, China

**Keywords:** overactive bladder, serum total bilirubin, a population-based study, risk assessment, NHANES

## Abstract

**Background:**

The relationship between serum total bilirubin (STB) concentrations and the risk of overactive bladder (OAB) remains uncertain. This study aims to explore the potential connection between STB and OAB.

**Method:**

We utilized data from the National Health and Nutrition Examination Survey (NHANES) database for the years 2001-2020. Weighted multivariate logistic regression and restricted cubic spline (RCS) analysis assessed the relationship between STB and OAB. Interaction analyses on subgroups were performed to validate the findings.

**Results:**

The study included 28,958 participants, with 5,313 identified as having OAB. The results from the fully adjusted models revealed an odds ratio (OR) of 0.98 (95% CI: 0.97, 0.99) and a statistically significant *P* value of less than 0.001 for the relationship between STB and OAB. Notably, individuals in the second and third tertiles exhibited significant differences compared to those in the lowest STB tertile, with respective odds ratios (*P* values) of 0.88 (0.04) and 0.80 (<0.001). RCS analysis indicated a non-linear association between STB and OAB (*P* for nonlinearity = 0.042), with an inflection point at 16.8 μmol/L. The association between STB and OAB exhibited consistency across various subgroups, except for stratification by age and diabetes status(*P* for interaction = 0.002 and 0.004, respectively), indicating a stronger correlation in individuals younger than 60 years or those without diabetes.

**Conclusion:**

These findings suggest an inverse association between STB concentrations below 16.8 μmol/L and the incidence of OAB. Bilirubin could potentially serve as an intervention or risk assessment tool for OAB in future studies.

## Introduction

Overactive bladder (OAB) is recognized by the International Continence Society as a chronic condition, featuring urinary urgency, often with frequent urination and nocturia, with or without urge incontinence, and in the absence of urinary tract infection or other overt pathology (1). OAB becomes more common with age, according to some epidemiological surveys, which estimate its prevalence to be between 16% and 19% (2, 3).

OAB and its symptoms have been linked to an increased prevalence of depression, considerably impacting individuals’ daily activities and social interactions (4). The healthcare expenses for patients suffering from OAB are more than 2.5 times those of patients without the condition, creating a substantial burden on both their families and social welfare (3, 5).

The etiology of OAB is considered multifaceted, involving neurogenic, myogenic, urotheliogenic, or integrative factors (6).the. Despite extensive research, the precise pathogenesis of OAB remains poorly understood, with previous studies suggesting that chronic inflammation and elevated oxidative stress levels may play a role in its development (7, 8).

Bilirubin, a tetrapyrrole ring structure pigment, is the final product of heme catabolism in the circulatory system (9). Bilirubin is recognized for its diverse physiological functions, including its antioxidant and anti-inflammatory properties (10, 11). Studies into Gilbert’s syndrome suggest that slightly elevated serum bilirubin levels are associated with a lower prevalence of chronic conditions such as cardiovascular diseases, certain cancers, and autoimmune or neurodegenerative diseases (12). To date, the evidence linking bilirubin levels and OAB is still relatively scarce. Identifying this association could provide novel insights for OAB patients. Thus, we explored the link between STB and OAB in a significant population, utilizing data from the NHANES (2001–2020).

## Methods

### Data source and study population

This cross-sectional analysis utilized data from the National Health and Nutrition Examination Survey (NHANES) cohorts. The NHANES protocols received approval from the ethics review board of the National Center for Health Statistics, with informed consent provided by all participants. Further information about NHANES is available on its official website.

**Figure 1:**
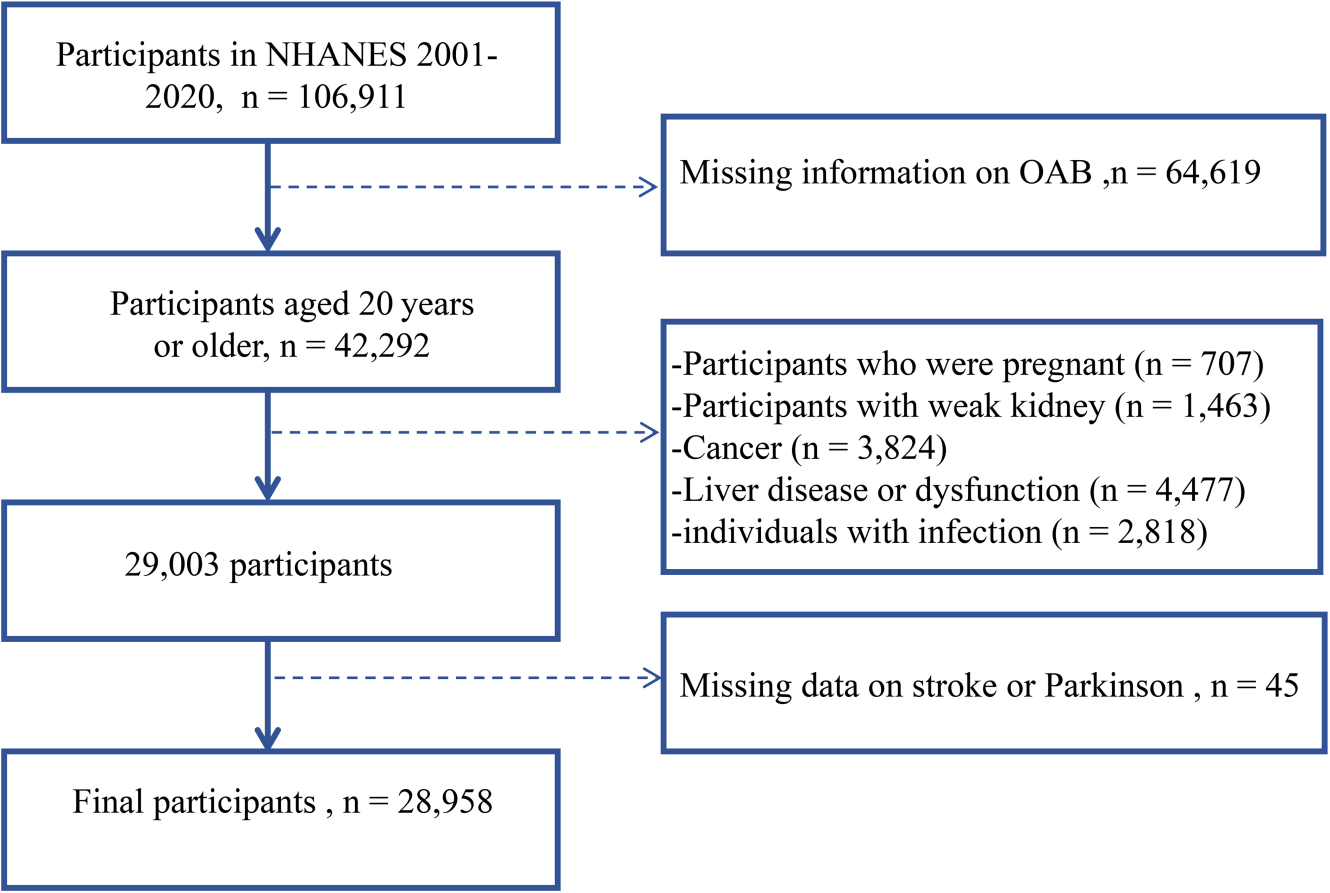
Flowchart of sample inclusion and exclusion criteria. Dotted lines indicate the exclusion process.

From the continuous NHANES cycles spanning 2001 to 2020, a starting pool of 106,911 participants was selected. Exclusions were applied for various reasons: 64,619 participants were excluded due to missing OAB data, 707 for pregnancy, 1,463 due to kidney issues, 3,824 for cancer, and 4,477 for liver dysfunction, encompassing both current and historical cases. Current liver disease criteria included elevated total bilirubin levels (>34.2 µmol/L), aspartate aminotransferase or Alanine aminotransferase levels (>80 IU/L), or a self-reported history. Historical liver disease was determined by participant affirmation of having been diagnosed with any liver condition (mcq160l). To minimize the influence of inflammation on the symptoms of overactive bladder (OAB), individuals with infections or inflammatory conditions, indicated by a whole blood leukocyte count exceeding 10×10^9/L (n = 2,818), were excluded from the study. Furthermore, participants without available data on stroke and Parkinson’s disease were also excluded (n = 45). Consequently, a total of 28,958 participants were deemed eligible for inclusion in this study. The selection process is illustrated in **Figure 1**.

### Diagnosis of OAB and detection of STB

Overactive bladder refers to an excessively active urination reflex, which is indicated by urge urinary incontinence (UUI) and frequent nighttime urination. The diagnostic criteria for OAB are based on existing literature reports (13). The data collected here was obtained from questionnaires filled out by trained research personnel during face-to-face interviews. Furthermore, OAB was assessed using the overactive bladder symptom score (OABSS). The cumulative Overactive Bladder Symptom Score for each subject was determined by combining scores for nocturia and urge incontinence. Participants with a total score of 3 or higher were categorized as having OAB.

The automated biochemical analyzer DXC800 (Beckman, USA) was used to measure STB concentration using the Jendrassik-Grof timed-endpoint Diazo method. During this assay, bilirubin combines with a diazo reagent and accelerators, including caffeine, benzoate, and acetate, to generate azobilirubin. The assay determines STB by measuring the absorbance change at 520 nm over a specific duration.

### Assessment of covariates

To evaluate the potential impact of factors that might influence the outcomes, variables identified as potential confounders were considered. These include age (in years), gender (male or female), ethnicity (non-Hispanic white, non-Hispanic black, Mexican American, or other), marital status (married/living with partner, widowed/divorced/separated, or never married), education level (less than high school, high school graduate, or beyond high school), poverty level (family income-to-poverty ratio below 1 or above 1). Alcohol consumption and smoking status were categorized into never, former, and current. Hypertension, diabetes mellitus(DM), Parkinson’s disease, and stroke were categorized as binary variables denoting absence or presence.

### Statistical analysis

We conducted a comprehensive statistical analysis adhering to NHANES analytic guidelines (https://wwwn.cdc.gov/nchs/nhanes/tutorials/module2.aspx). To mitigate the impact of directly deleting missing data on statistical results, we used the Jomo package for multiple imputations to handle missing covariates. Continuous variables were reported as mean ± standard error (SE) and categorical variables as number (percentage). Baseline characteristics between subjects with and without OAB were compared using unpaired Student’s t-test or weighted Mann-Whitney U for continuous variables and χ^2^ tests for categorical variables. The relationship between STB and OAB was explored through continuous and categorical analyses employing survey weight-adjusted logistic regression models. Furthermore, a weighted restricted cubic spline (RCS) was utilized to clarify the dose-response relationship between STB concentration and OAB risk. Moreover, subgroup analysis was conducted to evaluate the potential modifying effects of covariates on the STB-OAB relationship. All statistical analyses were conducted using R 4.2.3, with a significance level of *P* < 0.05 considered statistically significant. The **Supplementary Materials** shows the statistical results after the deletion of covariate missing data (n=1,928, 7.1%) (**Supplementary Tables S1, S2**, **Supplementary Figures S1, S2, S3**). The conclusions drawn from the two statistical methods are basically consistent, indicating that the relationship between STB-OAB is not affected by whether covariate missing data is imputed.

## Results

### Basic characteristics

**Table 1** displays the weighted distribution of characteristics across the study population. Significant differences in baseline characteristics were observed between the OAB and non-OAB groups. On average, individuals in the Non-OAB group were younger (mean ± SE, 44.33 ± 0.22 years) and had a lower Body Mass Index (BMI) (28.35 ± 0.08 kg/m^2^). This group also had a lower percentage of females (47.61%), individuals with diabetes mellitus (DM) (9.74%), and those with hypertension (29.98%) compared to the OAB group. Conversely, bilirubin levels were significantly higher in the Non-OAB group (11.14 ± 0.07 µmol/L) compared to the OAB group (10.17 ± 0.11 µmol/L). Additionally, compared to their counterparts, individuals in the OAB group were less likely to have received high school education (Beyond high school, 49.79%), be in a solitary status (widowed, divorced, separated, or never married, 40.93%), and have a history of smoking (either former or current smokers, 47.19%), Parkinson’s disease (1.75%), and stroke (6.34%). After removing individuals with missing covariates(n = 1,928, 6.6%), the number of subjects in the study population was 27,075, and the characteristics of different populations remained consistent (**Supplementaryay Table S1**).

**Table 1:** Basic characteristics of the study participants.

Characteristic	Total (n=28,958)	Non OAB (n=23,635)	OAB (n=5,313)	*P*-value
Age(years)	46.07 ± 0.22	44.33 ± 0.22	57.06 ± 0.33	< 0.0001
STB(μmol/L)	11.01 ± 0.07	11.14 ± 0.07	10.17 ± 0.11	< 0.0001
BMI	28.73 ± 0.08	28.35 ± 0.08	31.07 ± 0.14	< 0.0001
Gender(%)				< 0.0001
Female	14401 (49.71)	11211 (47.61)	3190 (62.93)	
Male	14557 (50.29)	12434 (52.39)	2123 (37.07)	
Ethnicity/Race(%)				< 0.0001
Non-Hispanic White	11318 (66.05)	9492 (66.89)	1826 (60.71)	
Non-Hispanic Black	6692 (11.46)	4997 (10.30)	1695 (18.82)	
Mexican American	4498 (8.74)	3706 (8.84)	792 (8.12)	
other	6450 (13.74)	5450 (13.97)	1000 (12.35)	
Marital status(%)				< 0.0001
Married/living with partner	17380 (64.28)	14597 (65.11)	2783 (59.07)	
Widowed/Divorced/Separated	5925 (16.53)	4145 (14.66)	1780 (28.37)	
Never married	5653 (19.19)	4903 (20.23)	750 (12.56)	
Education level(%)				< 0.0001
less than high school	6561 (14.46)	4813 (12.98)	1748 (23.78)	
High school/GED	6667 (23.31)	5347 (22.82)	1320 (26.43)	
Beyond high school	15730 (62.24)	13485 (64.21)	2245 (49.79)	
Smoking(%)				< 0.0001
Never	16993 (58.22)	14161 (59.10)	2832 (52.81)	
Former	6632 (24.07)	5127 (23.22)	1505 (29.53)	
Now	5315 (17.67)	4345 (17.68)	970 (17.66)	
Alcohol consumption(%)				< 0.0001
Never	4085 (10.78)	3175 (10.17)	910 (14.65)	
Former	3626 (10.78)	2667 (9.70)	959 (17.57)	
Now	21247 (78.44)	17803 (80.12)	3444 (67.78)	
DM(%)				< 0.0001
No	24235 (88.44)	20504 (90.26)	3731 (76.98)	
Yes	4723 (11.56)	3141 (9.74)	1582 (23.02)	
Hypertension(%)				< 0.0001
No	17947 (66.72)	15791 (70.02)	2156 (45.90)	
Yes	11011 (33.28)	7854 (29.98)	3157 (54.10)	
Parkinson(%)				< 0.0001
No	28728 (99.24)	23511 (99.40)	5217 (98.25)	
Yes	230 (0.76)	134 (0.60)	96 (1.75)	
Stroke(%)				< 0.0001
No	28013 (97.72)	23107 (98.36)	4906 (93.66)	
Yes	945 (2.28)	538 (1.64)	407 (6.34)	

BMI, body mass index; DM, diabetes mellitus; STB, serum total bilirubin.

### Multivariate analysis

The association between STB and OAB was assessed using weighted logistic regression analysis (**Table 2**). The crude model indicated that each one μmol/L increase in STB was linked to a 4% reduction in OAB risk (odds ratio, OR) = 0.96; 95% CI: 0.95−0.97; *P* < 0.0001). After adjusting for a subset of covariates (Model 1) and then for all covariates (Model 2), serum bilirubin levels remained an independent risk factor for OAB (OR 0.97, 95% CI 0.96-0.98, *P <*0.0001; OR 0.98, 95% CI 0.97-0.99, *P* < 0.001, respectively).

**Table 2:** Association between serum bilirubin and risk of OAB in multiple survey-weighted logistic regression models.

Bilirubin level	Crude model		Model 1		Model 2	
	ORs (95%CI)	*P*-value	ORs (95%CI)	*P*-value	ORs (95%CI)	*P*-value
Continuous variable	0.96 (0.95,0.97)	<0.0001	0.97 (0.96,0.98)	<0.0001	0.98 (0.97,0.99)	<0.001
Categorical variable						
T1 of STB	1(Ref)		1 (Ref)		1 (Ref)	
T2 of STB	0.83 (0.74,0.94)	0.003	0.84 (0.74,0.95)	0.01	0.88 (0.77,0.99)	0.04
T3 of STB	0.61 (0.55,0.68)	<0.0001	0.72 (0.63,0.81)	<0.0001	0.80 (0.71,0.90)	<0.001
*P* for Trend		<0.001		0.003		0.03

ORs, modds ratio; CI, confidence interval; Tertile 1 (≤8.55), Tertile 2 (8.55<STB ≤ 11.97), Tertile 3 (11.97<STB ≤ 34.2); No covariable was adjusted in Crude Model. Model 1 was adjusted for gender, age, and race/ethnicity and Model 2 was additionally adjusted for educational level, smoking status, alcohol consumption, body mass index, hypertension, diabetes, Parkinson, and stroke.

To further investigate the STB-OAB relationship, STB was categorized into tertiles, with the lowest tertile as the reference group (**Table 2**). In Model 1, the OR in the second tertile (T2) was 0.84 (95% CI, 0.74–0.95; *P*< 0.01) compared to the first tertile (T1). In Model 2, the OR for T2 remained significant (OR = 0.88; 95% CI, 0.77–0.99; *P* = 0.04). Consistency was observed in the T3 subgroup, with an OR of 0.72 (95% CI, 0.63–0.81; *P* < 0.0001) in Model 1 and an OR of 0.80 (95% CI, 0.71–0.90; *P* < 0.001) in Model 2. All *P* for trend were less than 0.01. The research conclusions of multiple model logistic regression remain consistent after excluding individuals with missing values in covariates (**Supplementary Table S2**).

### The detection of nonlinear relationships

**Figure 2:**
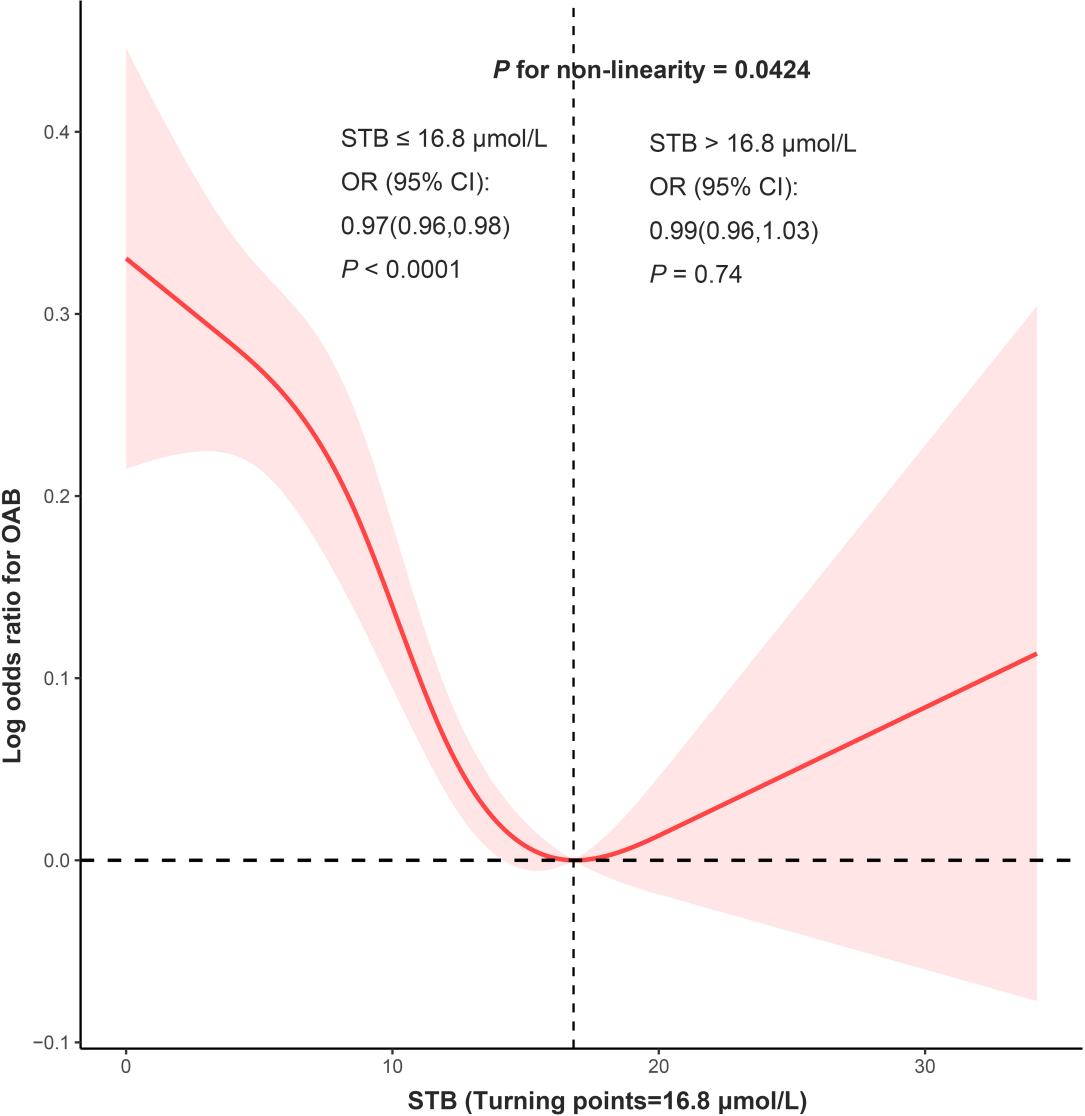
RCS analysis was adjusted for gender, age, and race/ethnicity, educational level, smoking status, alcohol consumption, body mass index, hypertension, diabetes, Parkinson, and stroke.

The RCS analyses aimed to address potential bias from assumed linearity and accurately evaluate dose-response relationships. The RCS indicated a non-linear relationship (*P* for nonlinearity=0.0424) between STB levels and OAB risk in Model 2. A threshold point was identified at 16.8 μmol/L (**Figure 2**). For STB levels ≤16.8μmol/L, the risk of OAB decreased with increasing STB (OR 0.97, 95% CI 0.96-0.98, *P* < 0.0001). Conversely, for STB levels >16.8μmol/L, no significant association was found between STB and OAB risk (OR 0.99, 95% CI 0.96-1.03, *P* = 0.74). After excluding individuals with missing covariate data, the overall trend of the RCS curve remains consistent, notwithstanding minor numerical variations. As illustrated in **Supplementary Figure S2**, for STB levels ≤16.5 μmol/L, the risk of OAB decreases with increasing STB, with statistical significance (P < 0.001).

### Stratified and interaction analyses

**Figure 3:**
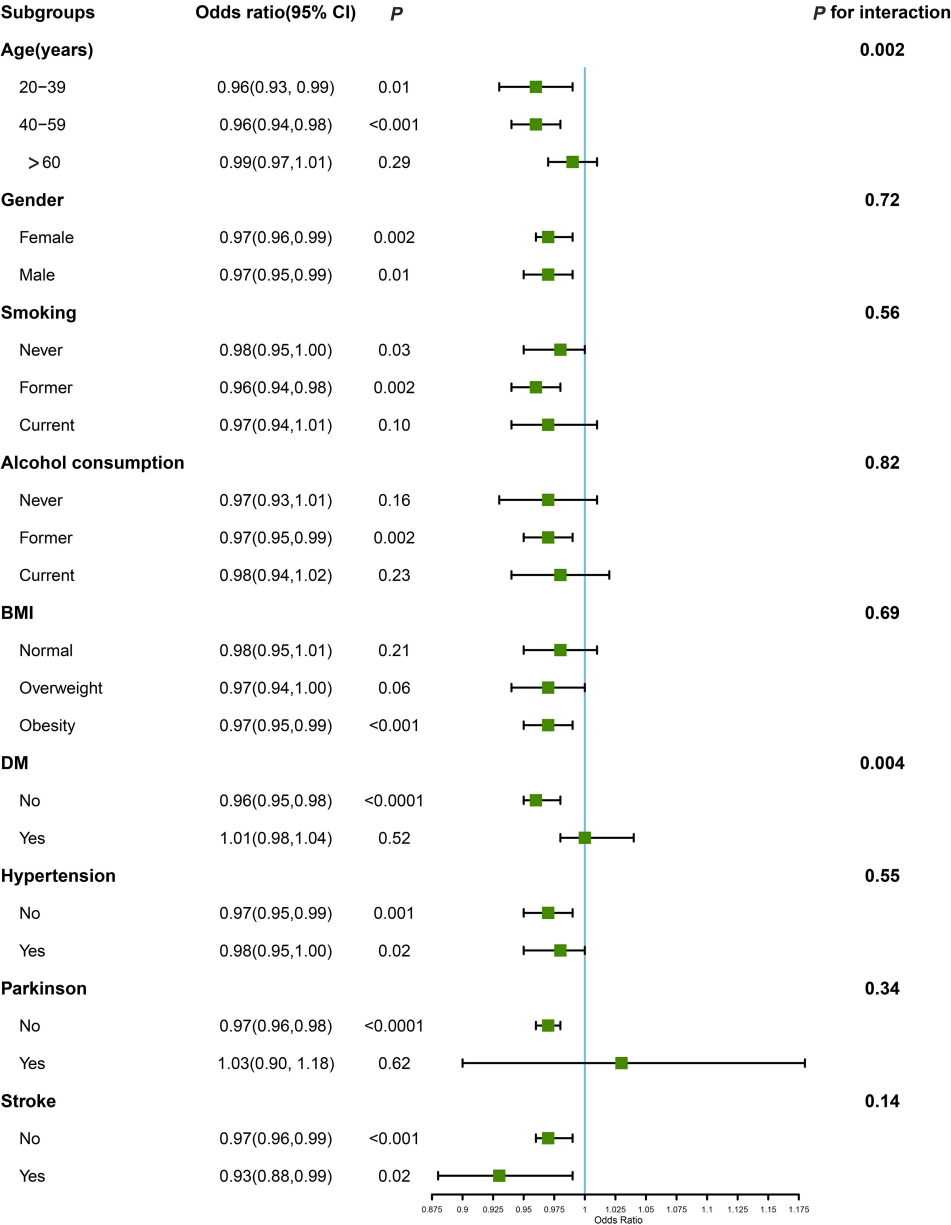
Associations between STB and OAB in subgroups. Estimates were adjusted for gender, age, and race/ethnicity, educational level, smoking status, alcohol consumption, body mass index, hypertension, diabetes, Parkinson, and stroke. Abbreviations: CI: confidence interval; BMI: body mass index.

To further elucidate the association between STB (≤16.8μmol/L) and OAB, we performed interaction analysis using model 2(**Figure 3**). The correlation between STB and OAB differed based on age and diabetes status (*P* for interaction = 0.002 and 0.004, respectively). The association between STB and OAB was more prominent in individuals younger than 60 years or those without diabetes. In contrast, this association did not reach statistical significance in individuals aged 60 years and older, or those with diabetes. Furthermore, no additional interactions were observed among other variables. After excluding individuals with missing covariate data, the correlation between STB and OAB continued to vary according to age and diabetes status (**Supplementary Figure S3**, P for interaction = 0.02 and 0.01, respectively).

## Discussion

This study represents a novel investigation into the relationship between OAB and STB concentrations utilizing the comprehensive dataset of the National Health and Nutrition Examination Survey (NHANES). Our findings reveal a negative dose-response correlation, wherein elevated STB levels below the identified threshold of 16.8 μmol/L are associated with a reduced likelihood of OAB. However, this association loses statistical significance beyond this threshold. These findings have important implications for risk evaluation and therapeutic interventions for individuals with OAB.

Studies concentrating on Asian populations have demonstrated that overactive bladder (OAB) symptoms affect 20% of individuals aged 40 years and older in countries such as China, Taiwan, and South Korea, with prevalence increasing with advancing age (14). Various factors, including age, body mass index (BMI), gender, diabetes mellitus, hypertension, Parkinson’s disease, as well as social and psychological factors, have been identified as contributors to OAB. Our results emphasize that comorbid conditions (e.g., diabetes, obesity, and hypertension) are significantly related to increased OAB symptomatology. Supporting our findings, another study demonstrated that age (OR = 2.26, 95% CI: 1.6-3), a higher BMI (OR = 2.6, 95% CI: 1.8-3.8), and the presence of comorbidities (OR = 2.6, 95% CI: 1.9–3.5) were significantly associated with an elevated risk of OAB (*P* < 0.001) (14).

Moreover, the prevalence of OAB is influenced by a combination of physical and psychosocial determinants. A comprehensive examination of forty-three scholarly articles demonstrated a significant association between depression and OAB in 26 studies, as well as a connection between anxiety and OAB in six studies (4). Our findings support the notion that marital status and educational attainment are correlated with OAB, as individuals who are married or cohabitating and those with higher levels of education (beyond high school) exhibit a decreased likelihood of experiencing OAB symptoms. This implies that maintaining a more stable psychological state may contribute to a reduction in the occurrence of OAB.

Recent findings highlight bilirubin’s multifunctional roles, including antioxidant activities (15), immunosuppressive effects (16), endocrine functions (17), and interconnected impacts, which may contribute to a decreased risk of chronic diseases (12, 18). However, the role of bilirubin in bladder-related diseases, particularly OAB, has been underreported (19). Given the potential for inflammation and oxidative stress to exacerbate OAB, bilirubin may play a protective role in mitigating these factors and slowing the progression of OAB (20, 21). Our study provides valuable insights by demonstrating a clear inverse relationship between bilirubin levels and the risk of OAB. We segmented bilirubin concentration into three tertiles (T1, T2, T3), uncovering that protection against OAB escalates with increasing bilirubin levels. To examine whether this protective effect progresses linearly, we utilized RCS analysis for a non-linear relationship. The findings indicate that the association between bilirubin levels and OAB is non-linear, with a notable inflection point at 16.5 μmol/L according to piecewise regression analysis. Below this threshold, OAB risk decreases with rising bilirubin levels. Above this point, the relationship shows no significant linear trend. This pattern mirrors outcomes from a cross-sectional study on the association between organophosphate exposure and OAB (22). Our stratified analysis, focusing on bilirubin concentrations under 16.8μmol/L, identified a pronounced negative relationship in individuals younger than 60, suggesting bilirubin as a potential therapeutic target for this age group. Furthermore, a significant disparity in the efficacy of bilirubin’s protective properties against OAB was noted between individuals with and without diabetes, with a lack of statistical significance in the correlation between STB levels and OAB in diabetic individuals. It is hypothesized that diabetic neuropathy may contribute to or exacerbate OAB symptoms, potentially negating the antioxidant and anti-inflammatory benefits of bilirubin. This suggests a need for further investigation into the underlying mechanisms involved.

Several limitations should be acknowledged. The retrospective cohort design of this study precludes inferring a causal relationship between bilirubin levels and OAB incidence. Moreover, the reliance on self-reported questionnaires within the NHANES for data collection on OAB, coupled with the absence of specific laboratory assessments such as urodynamics and cystoscopy, may compromise the precision of the statistical outcomes. Additionally, the database’s insufficiency in providing comprehensive details on recent medication usage, urinary calculi, and benign prostatic hyperplasia could potentially influence the validity of the conclusions drawn. The underlying mechanism of this association requires further elucidation in future research, and it is recommended that prospective, large-scale studies be conducted to validate the predictive significance of serum bilirubin levels.

## Conclusion

This study substantiates that elevated serum bilirubin levels, provided they do not exceed 16.8 μmol/L, exhibit an inverse correlation with the incidence of OAB in adults under the age of 60 who do not have DM. Consequently, bilirubin may be considered a potential therapeutic target for mitigating OAB risk in these populations. Future preventive and therapeutic strategies could involve increasing bilirubin levels or the intake of plant-derived pigments with properties similar to bilirubin.

## Data Availability

Publicly available datasets were analyzed in this study. This data can be found here: http://wwwn.cdc.gov/nchs/nhanes/default.aspx.
