# Supplementary figures and images for: Associations between serum total bilirubin and overactive bladder from the National Health and Nutrition Examination Survey
Source: Front Endocrinol (Lausanne). 2025 Jan 14;15:1421426. doi: 10.3389/fendo.2024.1421426 (PMC11772181; doi:10.3389/fendo.2024.1421426)

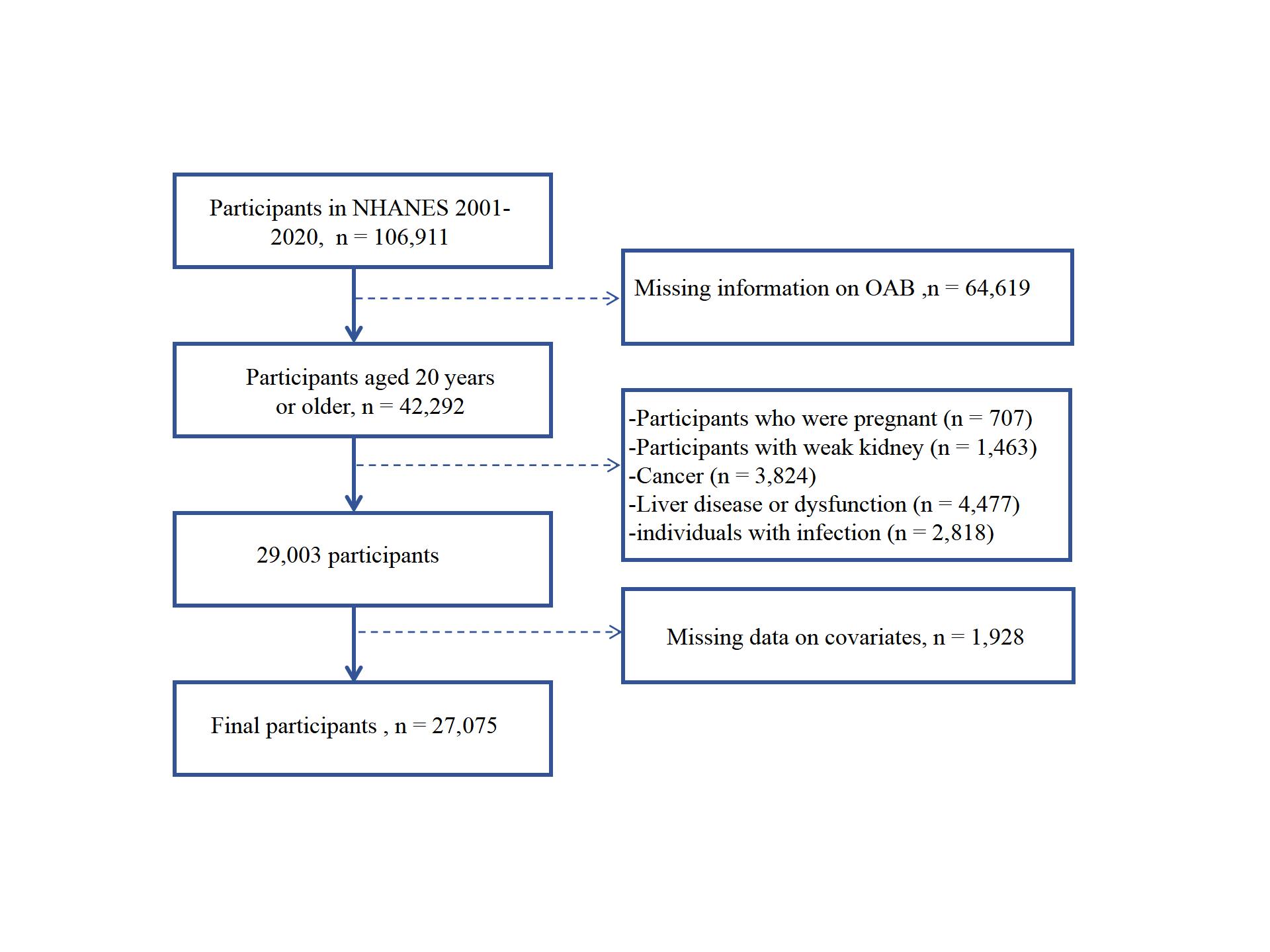

Supplement: Supplementary file 1 [file Image1.jpeg]

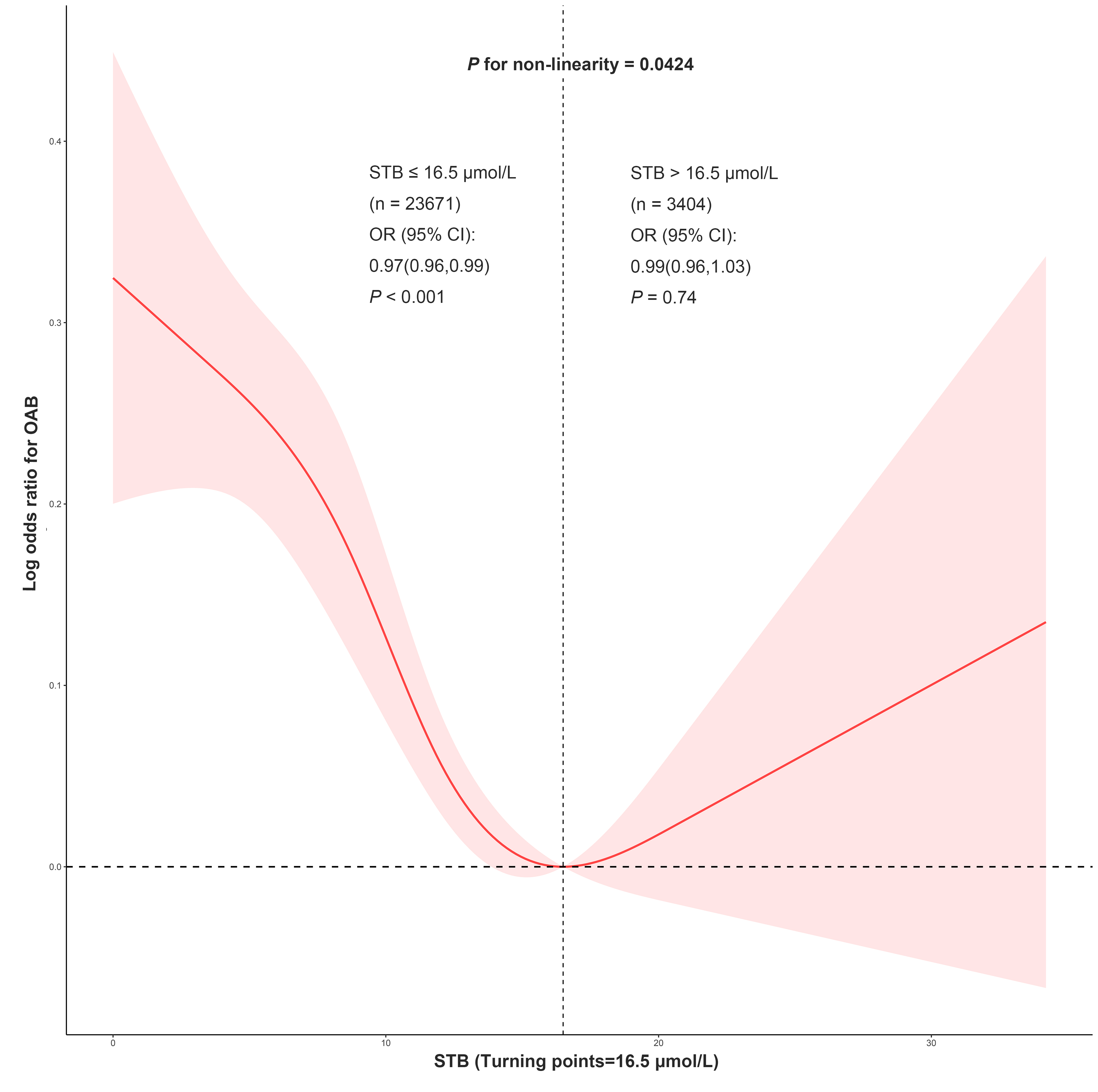

Supplement: Supplementary file 2 [file Image2.jpeg]

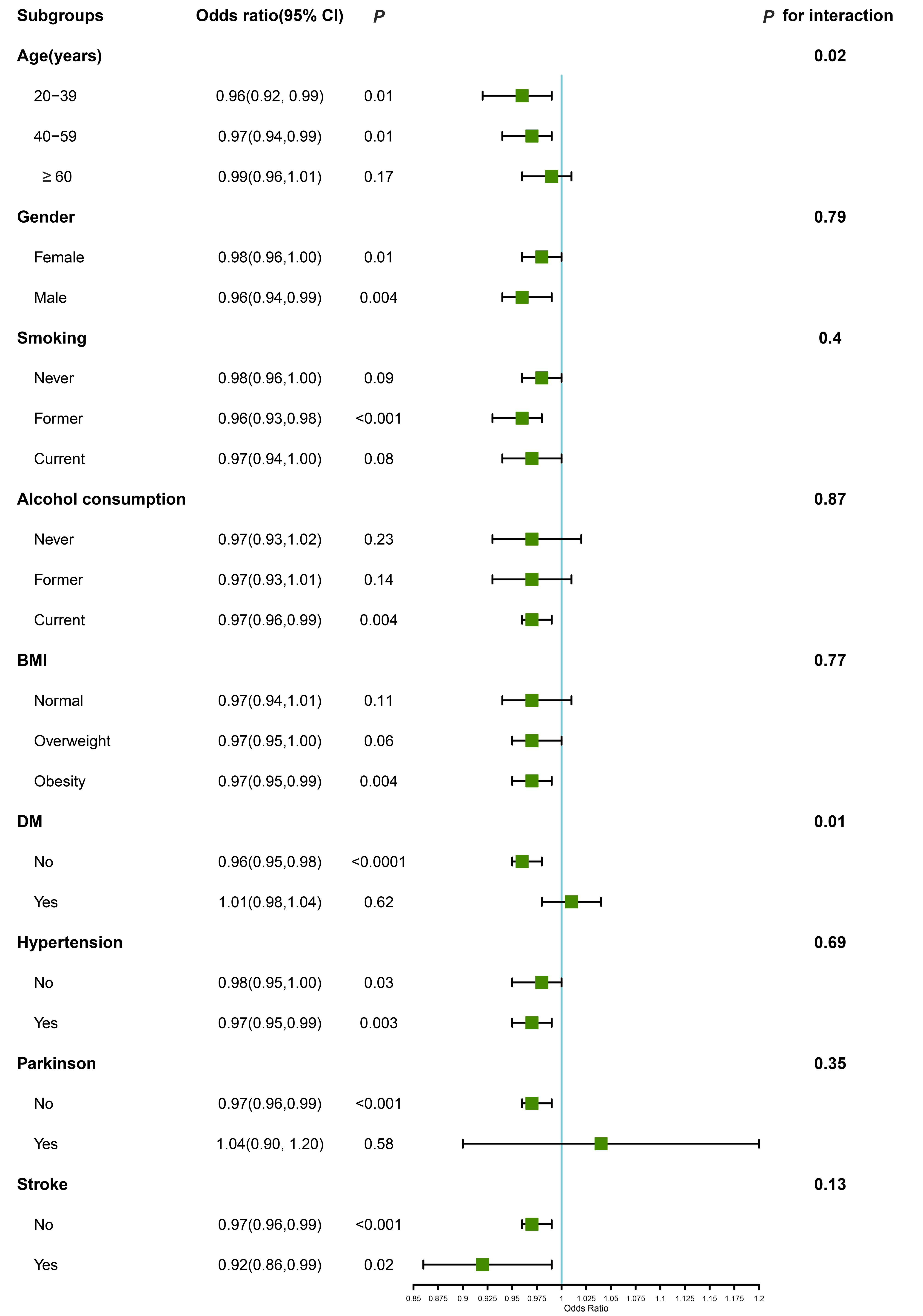

Supplement: Supplementary file 3 [file Image3.jpeg]
