# Supplementary material for: Associations between serum total bilirubin and overactive bladder from the National Health and Nutrition Examination Survey
Source: Front Endocrinol (Lausanne). 2025 Jan 14;15:1421426. doi: 10.3389/fendo.2024.1421426 (PMC11772181; doi:10.3389/fendo.2024.1421426)
Supplement: Supplementary file 4 [file Table1.docx]

SupplementaryTable 1 Basic characteristics of the study participants without missing values

| Characteristic | Total  (n=27,075) | Non OAB (n=22,334) | OAB (n=4,741) | *P*-value |
| --- | --- | --- | --- | --- |
| Age(years) | 45.67 ± 0.22 | 44 ± 0.23 | 56.66 ± 0.36 | < 0.0001 |
| STB(μmol/L) | 11.13 ± 0.07 | 11.25 ± 0.08 | 10.36 ± 0.12 | < 0.0001 |
| BMI | 28.68 ± 0.08 | 28.33 ± 0.08 | 31 ± 0.14 | < 0.0001 |
| Gender(%) |  |  |  | < 0.0001 |
| Female | 13484(49.71) | 10631(47.72) | 2853(62.81) |  |
| Male | 13591(50.29) | 11703(52.28) | 1888(37.19) |  |
| Ethnicity/Race(%) |  |  |  | < 0.0001 |
| Non-Hispanic White | 10656(66.38) | 9016(67.18) | 1640(61.10) |  |
| Non-Hispanic Black | 6171(11.35) | 4679(10.25) | 1492(18.64) |  |
| Mexican American | 4262( 8.77) | 3537(8.85) | 725(8.22) |  |
| other | 5986(13.50) | 5102(13.72) | 884(12.05) |  |
| Marital status(%) |  |  |  | < 0.0001 |
| Married/living with partner | 16343(64.52) | 13837(65.28) | 2506(59.50) |  |
| Widowed/Divorced/Separated | 5364(16.17) | 3798(14.37) | 1566(28.06) |  |
| Never married | 5368(19.31) | 4699(20.35) | 669(12.44) |  |
| Education level(%) |  |  |  | < 0.0001 |
| less than high school | 6030(14.19) | 4473(12.79) | 1557(23.41) |  |
| High school/GED | 6188(23.04) | 5026(22.58) | 1162(26.11) |  |
| Beyond high school | 14857(62.77) | 12835(64.64) | 2022(50.47) |  |
| Smoking(%) |  |  |  | < 0.0001 |
| Never | 15953(58.53) | 13433(59.42) | 2520(52.67) |  |
| Former | 6105(23.76) | 4772(22.92) | 1333(29.29) |  |
| Now | 5017(17.71) | 4129(17.66) | 888(18.04) |  |
| Alcohol consumption(%) |  |  |  | < 0.0001 |
| Never | 3788(10.68) | 2967(10.06) | 821(14.74) |  |
| Former | 3385(10.72) | 2511( 9.66) | 874(17.68) |  |
| Now | 19902(78.61) | 16856(80.28) | 3046(67.58) |  |
| DM(%) |  |  |  | < 0.0001 |
| No | 22854(88.87) | 19496(90.62) | 3358(77.36) |  |
| Yes | 4221(11.13) | 2838( 9.38) | 1383(22.64) |  |
| Hypertension(%) |  |  |  | < 0.0001 |
| No | 17011(67.32) | 15045(70.47) | 1966(46.55) |  |
| Yes | 10064(32.68) | 7289(29.53) | 2775(53.45) |  |
| Parkinson(%) |  |  |  | < 0.0001 |
| No | 26864(99.24) | 22209(99.39) | 4655(98.21) |  |
| Yes | 211( 0.76) | 125(0.61) | 86(1.79) |  |
| Stroke(%) |  |  |  | < 0.0001 |
| No | 26280(97.91) | 21880(98.51) | 4400(93.96) |  |
| Yes | 795( 2.09) | 454(1.49) | 341(6.04) |  |

Abbreviations: BMI, body mass index; DM, diabetes mellitus; STB, serum total bilirubin.
