# Supplementary material for: Associations between serum total bilirubin and overactive bladder from the National Health and Nutrition Examination Survey
Source: Front Endocrinol (Lausanne). 2025 Jan 14;15:1421426. doi: 10.3389/fendo.2024.1421426 (PMC11772181; doi:10.3389/fendo.2024.1421426)
Supplement: Supplementary file 5 [file Table2.docx]

Supplementary Table 2 Association between serum bilirubin and risk of OAB in multiple survey-weighted logistic regression models

| Bilirubin level | Crude model | | Model 1 | | Model 2 | |
| --- | --- | --- | --- | --- | --- | --- |
|  | ORs(95%CI) | *P*-value | ORs(95%CI) | *P*-value | ORs(95%CI) | *P*-value |
| Continuous  variable | 0.96(0.95,0.97) | <0.0001 | 0.97(0.96,0.98) | <0.0001 | 0.98(0.97,0.99) | 0.003 |
| Categorical  variable |  |  |  |  |  |  |
| T1 of STB | 1(Ref) |  | 1(Ref) |  | 1(Ref) |  |
| T2 of STB | 0.85(0.76,0.96) | 0.01 | 0.84(0.73,0.95) | 0.01 | 0.88(0.77,1.00) | 0.05 |
| T3 of STB | 0.64(0.57,0.72) | <0.0001 | 0.73(0.64,0.83) | <0.0001 | 0.81(0.71,0.92) | 0.002 |
| *P* for Trend |  | <0.0001 |  | <0.0001 |  | 0.002 |

ORs=odds ratio; CI=confidence interval; Tertile 1 (≤8.55), Tertile 2 （8.55<STB≤11.97 ), Tertile 3 （11.97<STB≤34.2 ); No covariable was adjusted in Crude Model. Model 1 was adjusted for gender, age, and race/ethnicity and Model 2 was additionally adjusted for educational level, smoking status, alcohol consumption, body mass index, hypertension, diabetes, Parkinson, and stroke.
